# The Interplay between Daptomycin and the Immune System

**DOI:** 10.3389/fimmu.2014.00052

**Published:** 2014-02-12

**Authors:** Theodoros Kelesidis

**Affiliations:** ^1^Department of Medicine, Division of Infectious Diseases, David Geffen School of Medicine, University of California, Los Angeles, CA, USA

**Keywords:** daptomycin, immunity, lipopeptide, immunomodulation, antibiotics

## Abstract

Antibiotics may have bacteriostatic or bactericidal effects but may also cause immunomodulation. Lipopeptides are known immunomodulators that interact with pattern recognition receptors such as Toll-like receptors in antigen presenting cells. Daptomycin is a novel lipopeptide antibiotic with a lipid moiety and unique structure that in the presence of divalent ions may directly interact with lipid membrane phospholipids, the major component of lipid membranes in immune cells. Daptomycin may also penetrate immune cells including neutrophils and macrophages. However, the possible immunomodulatory effects of daptomycin remain unknown. Understanding these effects is important to determine whether this agent can provide protection against infectious challenge through multiple mechanisms. Preliminary studies suggest that daptomycin may have minimal effects on cytokine production and may have synergistic immunomodulatory effects in combination with other immunomodulators. This review focuses on the hypothesis that daptomycin may also have immunomodulatory effects but further studies are needed to investigate this hypothesis.

## Introduction

The increasing antimicrobial resistance represents a challenge in treating infections (1, 2). The increased incidence of multidrug-resistant gram-positive pathogens such as methicillin-resistant *Staphylococcus aureus* (MRSA) (2) emphasizes the need for antimicrobials with different mechanisms of action (2). Daptomycin is a lipopeptide antibiotic that is bactericidal against gram-positive bacteria including MRSA (3). The Food and Drug Administration has approved daptomycin for the treatment of skin infections caused by gram-positive pathogens and for the treatment of *S. aureus* right-sided endocarditis and bacteremia (4). The exact mechanism of action of this lipopeptide antibiotic remains to be fully elucidated but its antimicrobial activity is entirely dependent on calcium. Although some antibiotics may cause immunomodulation (5–8) and lipopeptides are known immunomodulators (9–11), the possible immunomodulatory effects of daptomycin have been minimally investigated. In this review, the suggested mechanism of action and the possible immunomodulatory role of the lipopeptide antibiotic daptomycin are discussed.

## Daptomycin, a Prototype of the Acidic Lipopeptide Family

**Figure 1:**
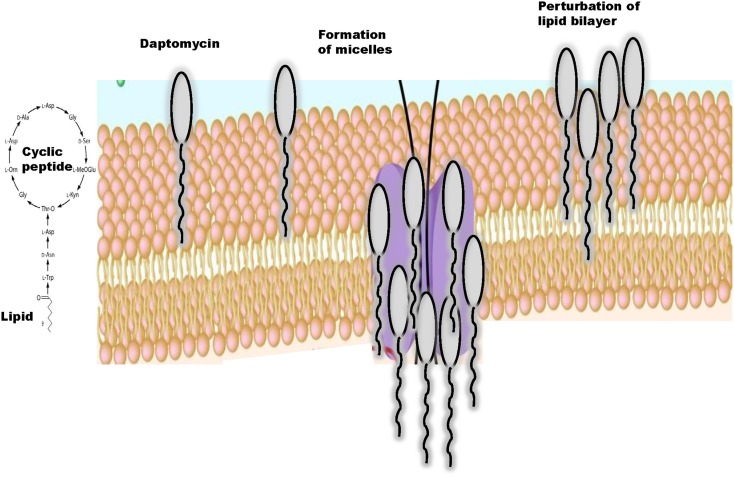
**Structure of daptomycin, a cyclic lipopeptide and interactions with the lipid bilayer**.

Daptomycin is a cyclic anionic lipopeptide antibiotic that was isolated from cultures of *Streptomyces roseosporus* (3, 12). This lipopeptide consists of 13 amino acid residues, which include 3 exocyclic d-amino acid residues (d-asparagine, d-alanine, and d-serine) and 3 non-proteinogenic amino acids including kynurenine (Kyn) that forms the macrolactone ring through an ester bond with Thr (13). *N*-decanoyl fatty acid chain, consisting of 10–13 carbon atoms, attached to the N-terminal Trp-1 is also characteristic (14, 15) (Figure 1). Thus, daptomycin has a lipophilic tail and a water-soluble core consisting of 13 amino acids. Daptomycin inherits a specific motif (DXDG), which is proposed to be involved in Ca^2+^ binding (16). Several other calcium-dependent lipopeptide antibiotics are known, including calcium-dependent antibiotic (CDA) (17–19) and they also share a long chain fatty acid attached to the cyclic core. The mechanistic and structural aspects of non-ribosomal product assembly of this lipopeptide have been previously reviewed in detail (20, 21).

## Mechanism of Action of Daptomycin

### The mechanism of action of daptomycin remains unclear but is highly dependent on divalent ions

Daptomycin acts bactericidal in a concentration-dependent manner but the mechanism of action of daptomycin is still under investigation (22). Daptomycin has a unique mode of antimicrobial action since free calcium ions at a concentration of 50 mg/l are required for the drug to become active and to bind to the bacterial cytoplasmic membrane (23). Currently, mechanistic studies largely focus on membrane insertion models and structures of daptomycin in the presence and absence of Ca^2+^ and it has been suggested that divalent cations promote formation of micelle by interacting with the negatively charged components of bacterial cell membranes such as phospholipids (14, 24) (Figure 1).

### Divalent ions induce conformational changes in daptomycin that facilitate interaction of daptomycin with the cytoplasmic membrane bilayer

Jung et al. (25) proposed a two-step mechanism of action of daptomycin that may explain how this lipopeptide interacts with cytoplasmic membranes. Initially in the presence of Ca^2+^, daptomycin binds to 1,2-dihexanoyl-sn-glycero-3-phosphocholine and experiences a minor conformational rearrangement (14, 26). This process increases amphipathicity of daptomycin, decreases its charge, allows daptomycin to interact with neutral or acidic membranes, and facilitates oligomerization (25) and micelle formation. In a second step, daptomycin interacts with the acidic phospholipids in the presence of Ca^2+^ and a major conformational change allows daptomycin to insert into bilayer membranes with acidic character (27, 28). In this model, the lipid tail promotes the formation of micelles, which are vehicles to allow daptomycin to insert into the bilayer (27), depolarize the membrane leading to cell death (14, 22, 25) (Figure 1). Thus, similarly to cationic peptides, the mechanism of action of daptomycin may involve multiple targets (29).

### Daptomycin directly interacts with phospholipids, a component of lipid membranes

Daptomycin can insert into artificial lipid vesicles and the membrane of gram-positive bacteria (22, 25, 30). Daptomycin contains two aromatic lipophilic residues (Trp-1 and Kyn-13) (30, 31) that upon their insertion in the phospholipid membrane becomes less polar (22, 25, 30). Daptomycin directly interacts with cytoplasmic membranes without requiring any receptors (22, 25, 30) and can insert into membrane vesicles composed of phosphatidylcholine (PC) and phosphatidylglycerol (PG) (25, 30). It has also been shown that daptomycin directly interacts with pulmonary surfactant, which sequesters and inhibits the antibiotic. Surfactant is composed mainly of dipalmitoylphosphatidylcholine and PG and only low levels of cholesterol and sphingolipids contrary to most eukaryotic membranes (32). Surfactant is also ~10% negatively charged PG, a major component of the gram-positive plasma membrane (33), which significantly enhances insertion of surfactant into the membrane (25). The major conformational change of daptomycin that leads to an increased perturbation of the cytoplasmic membrane is dependent on interaction with the bacterial acidic phospholipid, phosphatidyl glycerol (34). Although binding of daptomycin to bilayers containing acidic lipids primarily involves electrostatic interactions, presumably through the Ca^2+^-induced electrostatic bridging of the acidic amino acids in daptomycin with the acidic phospholipid head groups, the binding of daptomycin to neutral bilayers may occur through a combination of electrostatic and hydrophobic interactions (25, 35).

## Lipopeptides Like Daptomycin may Directly Interact with the Lipid Membranes of Immune Cells but the Possible Immunomodulatory Effects of Daptomycin Remain Unknown

### In the presence of calcium, daptomycin interacts with phosphatidylcholine, the major component of lipid membranes in eukaryotic cells

Cell membrane lipids in eukaryotes consist mainly of glycerophospholipids (GPLs), which are the major structural lipids in cellular membranes (36, 37). Although the major conformational change of daptomycin is dependent on interaction with the bacterial acidic phospholipid, phosphatidyl glycerol, in the presence of calcium daptomycin may directly interact with PC, which is the main bilayer-forming lipid in eukaryotic cells including immune cells (22, 25, 30). However, no studies have investigated the degree of interaction of daptomycin with lipid membranes of immune cells and further studies need to elucidate this possible interaction based on the known immunomodulatory activity of other lipopeptides.

### Lipopeptides are known adjuvants that may enhance immune responses

Adjuvants are substances that enhance the immune response in a variety of ways including activation of pattern recognition receptors (PRRs) on antigen presenting cells (APCs), which may result in the upregulation of proteins, such as co-stimulatory molecules and MHC class I and II (38–42). Most adjuvants are not physically associated with antigens and APCs could be activated by the adjuvant without taking up the antigen. Lipid-based adjuvants composed of lipopeptides, phosphatidylethanolamine (PE), PC, liposomes, lipid A (41, 42), muramyl dipeptide derivatives (38–40, 42) have previously been shown to exert immunopotentiating effects and increase immune responses in vaccines often through Fc receptor-mediated uptake and MHC class II presentation of antigens (42–47).

### Most lipopeptides interact with toll-like receptors

Lipopeptide adjuvants may form heterodimers with PRRs like Toll-like receptors (TLRs) that are present on APCs including macrophages and dendritic cells (9–11). Recognition by TLRs leads to activation of innate immunity through upregulation of co-stimulatory molecules and induction of inflammatory cytokines. The discovery of TLRs and their role in lipopeptide recognition contributed significantly to our understanding of how lipopeptides may interact with the immune system (48). Among several hypotheses, including prevention of enzymatic peptide degradation, it has been suggested that the lipid moiety of lipopeptides may be able to incorporate into cell membranes and deliver peptide epitopes into the APCs (48, 49). Several lipopeptides impart this self-adjuvanting activity by signaling via Toll-like receptor 2 (TLR2).

### The structure of lipopeptides determines their immunopotentiating effects

Lipopeptides consist of short structures of amino acids linked to fatty acids via ester or amide bonds and the acyl chains are often heterogeneous in terms of their length and degree of saturation. Recent understanding of how the lipid component of lipopeptides confers their activity as adjuvants could form the basis for vaccine development against numerous diseases. Synthetic lipopeptides have been used to determine the contribution of the lipid portion of lipopeptide to TLR2 specific recognition (9, 50). Stereochemical properties of the lipid moiety were found to influence aggregation behavior of the TLRs (51, 52). The activity of lipopeptides is not only influenced by the number and type of fatty acids present (9) but also on the position of the lipids (53). Lipopeptides may form heterodimers with TLRs that are stabilized by hydrogen bonding and hydrophobic interactions and may promote signaling (54). However, although daptomycin is a lipopeptide that has acyl groups and lipid moiety that could theoretically interact with PRRs like TLRs, this remains to be shown experimentally and the possible immunomodulatory effects of daptomycin have been minimally studied.

## The Interplay between Daptomycin and Immunity

### Antibiotics may have immunomodulatory effects

In addition to their antimicrobial activity, selected classes of antibiotics, like the macrolides, fluoroquinolones, oxazolidinones, and fosfomycin are increasingly recognized to exert immunomodulatory effects (5–8). Those antibiotics show large volumes of distribution, are able to modulate cytokines, may accumulate intracellularly in human blood and body cells to a varying extent, potentially allowing them to interact with DNA and affecting mRNA and protein synthesis (5–8). These immunomodulatory effects of antimicrobials further emphasize the need to elucidate the interplay between antibiotics and immunity, which may set the basis for extended uses of certain antimicrobials especially in the era of increased antimicrobial resistance. However, the possible immunomodulatory effects of daptomycin remain unclear.

### Daptomycin may penetrate into immune cells like neutrophils and macrophages

Antibiotics may interact with bacteria intracellularly in phagocytes (55, 56). Daptomycin may penetrate into human neutrophils and thus may be effective in killing intracellular bacteria (57). In one study, daptomycin had a 60% penetration to neutrophils (57). In another study, daptomycin had potent intracellular antibacterial effects against intracellular *S. aureus* in human monocyte-derived macrophages (58). Two studies have demonstrated that intracellular daptomycin activity in macrophages depends on the concentration of the extracellular antibiotic and the duration of the exposure to that concentration (55, 58). Thus, daptomycin may penetrate into immune cells but the significance of this penetration with regards to immunomodulation remains to be determined.

### Effects of daptomycin on cytokine production and innate immunity

Antibiotics may affect the overall balance of pro- and anti-inflammatory cytokines. The ability of daptomycin to affect pro-inflammatory cytokines was determined in one study by utilizing an established whole blood *in vitro* model (59). Thallinger et al. found that the addition of daptomycin at a therapeutically relevant concentration of 40 μg/ml in an experimental model of human endotoxemia had no effect on cytokines such as interleukin-1β (IL-1β), IL-6, and tumor necrosis factor alpha (TNF-α), neither on the mRNA nor on the protein levels (59). In the experimental *in vitro* study of Pichereau et al. on peripheral blood mononuclear cells, many different antibiotics including daptomycin, tended to reduce production of cytokines after toxin exposure (60). In the *in vitro* study of English et al., exposure of *S. aureus* isolates to daptomycin alone or in combination with vancomycin or oxacillin reduced macrophage inflammatory responses such as tumor necrosis factor secretion and accumulation of inducible nitric oxide synthase protein (compared with vancomycin or oxacillin alone) (61). The lack of any significant immunomodulatory effect of daptomycin observed in these preliminary studies may be explained by its very low volume of distribution. Although daptomycin has been shown to penetrate into immune cells (55–58), it remains to be determined whether the penetration of daptomycin into human cells is clinically significant because of the very hydrophilic, water-soluble core of this antibiotic. However, the uptake of daptomycin into mononuclear cells, which, beside the neutrophils, are mainly responsible for cytokine production and release, occurs predominantly via phagocytosis and pinocytosis. The penetration of daptomycin into the cell’s cytosol and nucleus is required for daptomycin to exert immunomodulatory effects on the human DNA (57, 58). Thus, daptomycin may be less likely to affect levels of cytokines probably due to high affinity of daptomycin to bacterial cytoplasmic membrane and its low potential to penetrate into human cells (59). Further studies are needed to determine the effects of daptomycin on cytokines. In one animal study in Balb/c mice, daptomycin did not affect humoral and cell-mediated immune responses such as polymorphonuclear phagocytic activity but the concentrations of daptomycin used were not representative for the concentrations used in humans after administration of standard dosages (62). Thus, it is important that in all the *in vitro* and *in vivo* studies testing immunomodulatory effects of antimicrobial agents, the concentrations of daptomycin should be representative for the concentrations used in humans after administration of standard dosages. In addition, the use of whole blood instead of peripheral blood mononuclear cells, as frequently done in many *in vitro* studies may reflect a more realistic clinical situation than the use of isolated, selected immunocompetent blood cells.

### Daptomycin may have synergistic immunomodulatory effects in combination with other immunomodulators like vitamin E

Vitamin E intake is associated with increased antimicrobial resistance (63–65). Current data revealed immunomodulatory properties of vitamin E also in human peripheral mononuclear cells altering cytokine production [interleukin (IL)-2, IL-8, and IL-17] in part by stimulating production of cyclic adenosine monophosphate (cAMP) (66). In an animal model, administration of the immune enhancer vitamin E before infecting 100 wounds by MRSA improved later the efficacy of daptomycin (67). Additionally, Pierpaoli et al. found that immune modulation was related to the antimicrobial effect of vitamin E with or without daptomycin (65). Gr-1+ cells and CD49b+ cells significantly increased in mice treated with vitamin E while daptomycin alone did not affect any of the leukocyte populations compared to control infected animals (65). Vitamin E plus daptomycin significantly increased CD49b+ cells compared with control infected animals or mice treated with vitamin E alone (65). Thus in animals treated with vitamin E with or without daptomycin, immunological changes such as modulation of natural killer (NK) cell activity and changes in leukocytes were associated with significant antibacterial activity (65). Further studies are needed to determine the possible immunomodulatory effects of daptomycin in combination with vitamins in humans (65).

### Daptomycin and NK cell activity

In one study, it was shown that administration of vitamin E plus daptomycin was associated with modulation of NK cell activity compared to controls (65). The groups given vitamin E plus daptomycin had significant increase in NK activity and cytotoxicity compared to vitamin E only or untreated animals suggesting an association of the antimicrobial effects with the immune modulation induced by the combination of daptomycin with vitamin E (67). Daptomycin alone did not significantly enhance NK activity compared with untreated infected mice. NK cells may bridge innate and adaptive immune responses and some of them respond to a variety of lipid antigens (68). Further studies are needed to understand the possible interaction between the lipid moiety of lipopeptide antibiotics and NK cells (65).

## Conclusion

**Figure 2:**
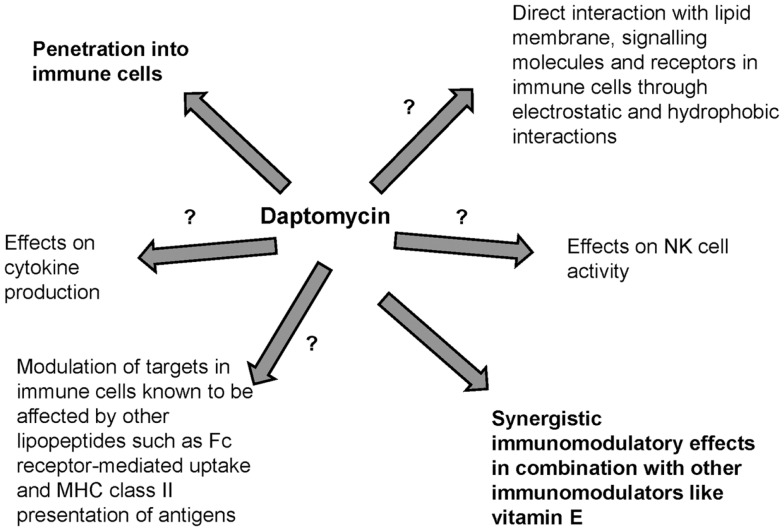
**Possible immunomodulation related to daptomycin**. Immunological effects of lipopeptides have previously been described but the interplay of daptomycin with immunity has been minimally studied.

Antibiotics may have bactericidal effects but may also cause immunomodulation. Lipopeptides are known adjuvants and immunomodulators that interact with PRRs such as TLRs in APCs. Daptomycin is a novel lipopeptide antibiotic that is largely water-soluble with a low volume of distribution but its lipid moiety and unique structure in the presence of divalent ions contribute to direct interaction with lipid membrane phospholipids. Although daptomycin preferentially interacts with acidic phospholipids that are present in the lipid membrane of gram-positive pathogens, it may also directly interact with human surfactant and PC, the major component of lipid membranes in immune cells. Daptomycin may also penetrate immune cells including neutrophils and macrophages. However, the possible immunomodulatory effects of daptomycin remain unknown (Figure 2). Preliminary studies suggest that daptomycin may have minimal effects on cytokine production and may have synergistic immunomodulatory effects (such as effects on NK cell activity) in combination with other immunomodulators. Understanding whether daptomycin may be an immunomodulatory agent is important to optimize the clinical use of this agent, especially in the era of increased antimicrobial resistance. Further studies are needed to investigate the interplay between daptomycin and immunity.

## Conflict of Interest Statement

The author declares that the research was conducted in the absence of any commercial or financial relationships that could be construed as a potential conflict of interest.
